# Mucopolysaccharidosis IIID and Beta‐Mannosidosis in Brazilian Anglo‐Nubian Goats: Molecular and Genealogical Insights for the Development and Implementation of a Genetic Disease Eradication Program

**DOI:** 10.1002/age.70107

**Published:** 2026-04-22

**Authors:** Flávia Caroline Moreira Bezerra, Lívia Samaniely Batista Pereira Belli, Joane Maíra Cavalcante Braga Novais, Adriana Mércia Guaratini Ibelli, Cássia Regina Oliveira Santos, Gisele Veneroni Gouveia, Alexandre Tadeu Mota Macedo, João José de Simoni Gouveia

**Affiliations:** ^1^ Center for Open Access Genomic Analysis (CALAnGO) Federal University of Vale of São Francisco (Univasf) Petrolina Pernambuco Brazil; ^2^ Postgraduate Program in Veterinary Sciences in Semiarid Federal University of Vale of São Francisco (Univasf) Petrolina Pernambuco Brazil; ^3^ Embrapa Pecuária Sudeste São Carlos São Paulo Brazil; ^4^ University Veterinary Clinic Federal University of Vale of São Francisco (Univasf) Petrolina Pernambuco Brazil

**Keywords:** animal breeding, genetic diseases, lysosomal storage diseases, molecular diagnostic, pedigree analysis, small ruminants

## Abstract

Beta mannosidosis and Mucopolysaccharidosis IIID are two autosomal recessive lysosomal storage diseases identified in Anglo‐Nubian goats. Even though they are well characterized from the clinical and molecular point of view, there is a gap in studies aiming to understand distribution and dissemination risk in goat populations throughout the world. Considering this, we developed probe‐based qPCR tests and combined it with pedigree analysis to deepen the knowledge related to the distribution of both mutations in Brazilian Anglo‐Nubian population. This study revealed, for the first time, that the mutation associated with Mucopolysaccharidosis IIID is segregating in our country, expanding the knowledge of the worldwide distribution of this disease‐associated allele. Additionally, the combination of genotyping and pedigree analysis enabled the estimation of additional high‐confidence putative genotypes, contributing to the reduction of genotyping costs and highlighting a putative pathway associated with the introduction/dissemination of the Mucopolysaccharidosis IIID‐associated allele in Brazil. These results can be considered as an initial basis for the proposition of a program to control/eradicate mutations associated with genetic diseases in Brazilian goat populations.

Beta mannosidosis and Mucopolysaccharidosis IIID are autosomal recessive inborn errors of metabolism described in Anglo‐Nubian goat breed related to neurological signs that can evolve to death in affected individuals (Lovell and Jones 1983; Thompson et al. 1992; Cavanagh et al. 1995; Friderici et al. 1995; Leipprandt et al. 1996; Lovell et al. 1998; Huynh et al. 2011; Smith and Sherman 2009; Nicholas et al. 2024).

These diseases are well characterized from the clinical and molecular point of view and considering the importance of Anglo‐Nubian breed worldwide (Stemmer et al. 2009), it is interesting to pinpoint that there is an enormous gap in studies aiming to describe the occurrence of affected animals throughout the world. This can be related to confounding and unspecific clinical signs along with the fact that the use of molecular techniques as complementary diagnostic methods is not a reality for professionals involved in small ruminant production in developing countries (Leipprandt et al. 1995, 1996; Gedik and Kavuncu 2021).

Therefore, the present study developed and validated an accurate, fast, and low‐cost method to identify mutations associated with Beta mannosidosis and Mucopolysaccharidosis IIID in caprine species and aimed to contribute to the comprehension of the distribution of both mutations in Anglo‐Nubian goat breed to serve as basis for the proposition of strategies to avoid the emergence of affected individuals.

DNA samples from two hundred ninety‐five (*n* = 295) individuals from Anglo‐Nubian goat breed belonging to 13 herds from the states of Pernambuco, Bahia, Ceará and Piauí (Brazil) were used in this study (Table S1).

Primers and Taqman‐MGB probes were designed using public reference sequences for caprine *GNS* (NC_030812.1) and *MANBA* (NC_030813.1) genes deposited in NCBI (Table S2). Additionally, two synthetic DNA fragments were synthesized and used as genotyping controls. Sequencing primers were designed (Table S3), and samples were submitted for Sanger sequencing to validate the genotyping procedure.

Genealogical information was retrieved from the Brazilian Goat Breeders Association database and pedigree visualization, and analysis was performed using Endog v4.8 (Gutiérrez and Goyache 2005) and R packages Kinship2 (Sinnwell et al. 2014), Pedigree (Coster 2013), and Genlib (Gauvin et al. 2015). Geneprob software was used to estimate the genotype probabilities from ungenotyped individuals (Kerr and Kinghorn 1996).

Genotyping results obtained in this study can be considered reliable, since it included synthetic DNA fragments as reaction controls (Figures S1 and S2) and the comparison of genotypes identified by real time PCR and Sanger sequencing showed a perfect match (Figures S3–S6). It is important to consider that the number of sequenced samples in this study can be considered low (mainly for the MANBA gene), and increasing the number of sequenced individuals certainly will enhance confidence in the real time PCR genotyping procedure. Despite this, the method proposed can be applied in a screening program aiming to identify causal mutations associated with genetic diseases in goats.

Despite the fact that high throughput methods are emerging as interesting alternatives to perform population screening in domestic species (Anderson et al. 2022; Cole et al. 2025; Irish Cattle Breeding Federation 2025), and mutations associated with genetic defects are also available in commercial livestock genotyping platforms (NEOGEN Australasia 2025), the use of real time PCR genotyping can be considered a feasible strategy for small ruminants, since it is cheap, fast and accurate (Clavijo et al. 2010).

Genotyping revealed sixteen (16/295) animals heterozygous for the mutation associated with Mucopolysaccharidosis IIID (CT) and two hundred seventy‐nine (279/295) individuals homozygous for the wild allele (CC). For the mutation associated with Beta‐Mannosidosis, it was observed only individuals homozygous for the wild allele (GG).

To the best of our knowledge, our study is the first attempt to identify causal mutations associated with Mucopolysaccharidosis IIID and Beta‐Mannosidosis in Anglo‐Nubian herds in Brazil and, although neither disease has been diagnosed in this country to date, it can be shown that screening for known mutations associated with recessive genetic diseases can reveal deleterious alleles segregating in our country.

Heterozygous for Mucopolysaccharidosis IIID were identified in eight out of 13 herds, revealing the presence of the mutation in three out of four states studied (Table S4).

The observed allele frequency for the Mucopolysaccharidosis IIID associated allele in this study (*n* = 295) was 2.703% and the expected genotype frequencies assuming the Hardy–Weinberg equilibrium were 94.668%, 5.259% and 0.073% for “CC”, “CT” and “TT”, respectively. Considering this, our study expands knowledge of the worldwide distribution of the disease associated allele, previously identified only in North America (Hoard et al. 1998; Clavijo et al. 2010; Mora‐Navarro et al. 2024). These results highlight the importance of molecular screening studies for mutations previously described in additional breeds and countries in which they were previously undescribed (Donner et al. 2018; Cole et al. 2025).

Also the fact that there is no description, to date, of affected individuals in Brazil suggests that it should be important to start a program aiming to disseminate information related to the disease to veterinary professionals and goat breeders to enable them to include the possibility of this disease in diagnostic procedures of individuals presenting neurological signs (Thompson et al. 1992; Smith and Sherman 2009).

Considering that published studies may not reflect real distribution of the causal mutation associated to Mucopolysaccharidosis IIID in Anglo‐Nubian populations in the United States and Mexico because of the limited number of individuals/herds genotyped, this data can be used as an initial effort to better understand the risk of dissemination of this mutation in these countries. In the same way, our results can suggest that the introduction process of the Anglo‐Nubian breed in Brazil and Mexico may be a factor influencing smaller frequencies of the mutation observed when compared with the U.S. (Hoard et al. 1998; Clavijo et al. 2010; Mora‐Navarro et al. 2024).

The absence of individuals harboring the causal mutation associated with Beta‐Mannosidosis in our study must also be interpreted cautiously, considering that the number of genotyped animals is limited and this study only included herds located in the Brazilian Northeastern region. This can be interpreted as a weakness of our study, since the limited number of sampled individuals can, by chance, prevent the identification of heterozygous individuals. It is interesting to note that there aren't studies describing the frequency and distribution of the mutation in the Anglo‐Nubian breed (Jonest and Dawsonl 1981; Leipprandt et al. 1996). Considering this, an effort to extend the genotyping of individuals, including more herds and lineages, could enable a better comprehension of the real importance of this mutation to the worldwide Anglo‐Nubian population.

Genealogical analysis revealed a pedigree with eleven generations and 1363 individuals. Most individuals (93.32%) presented inbreeding coefficients at zero or near zero (< 1.00%), 4.92% presented low (1.00%–12.50%), 1.32% presented moderate (12.50%–25.00%), and only 0.44% presented high (> 25.00%) F values.

The kinship matrix was used to identify possible clusters of heterozygous (Figure 1) and the pattern observed was used to group individuals into clusters to perform MRCA analysis that identified four clusters of individuals sharing common ancestors.

**FIGURE 1:**
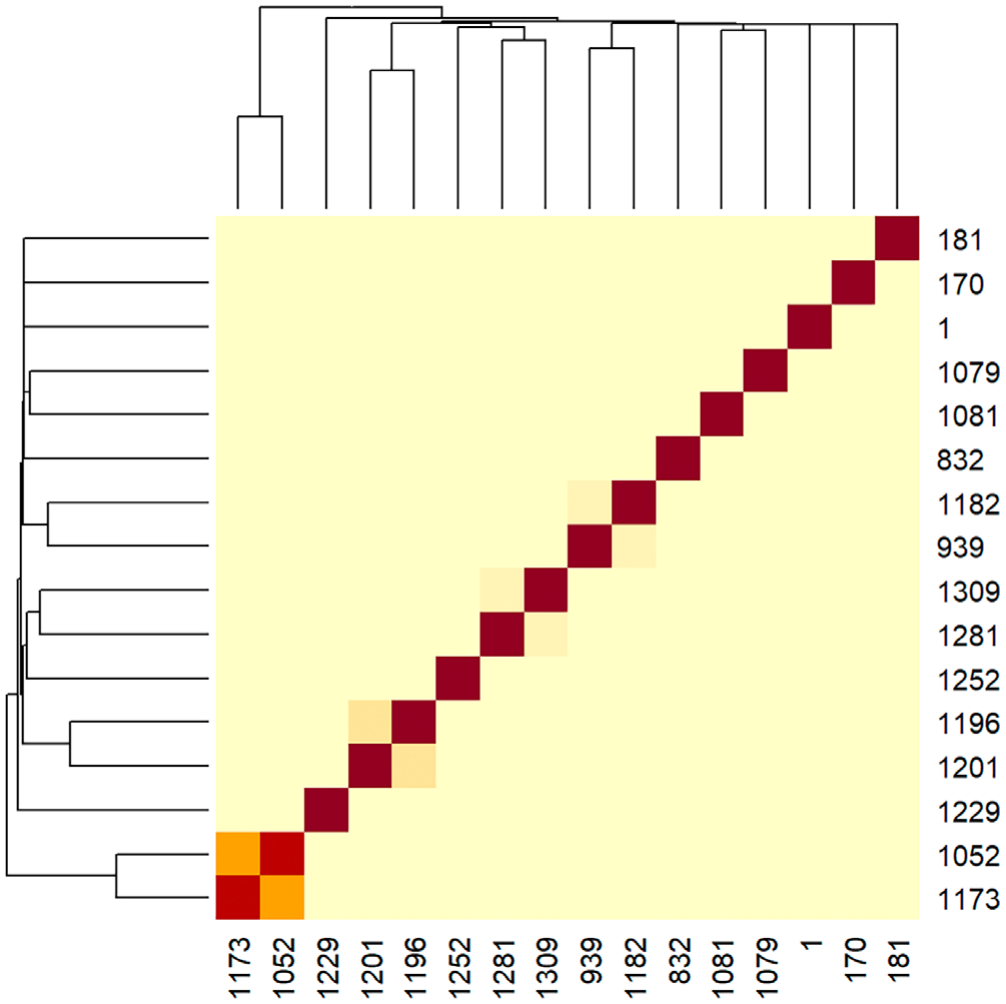
Heatmap of the kinship coefficients from heterozygous animals for the mutation associated with Mucopolysaccharidosis IIID identified in this study.

Three clusters, each one grouping two individuals (Table S5 and Figures S8–S10). In the first moment, it was not possible to identify clearly a historic pattern from this data. Interestingly, one cluster grouped six heterozygote individuals (Table S5 and Figure S7) originating from four different herds located in three different Brazilian states (Pernambuco, Bahia, and Ceará). This cluster allowed the identification of one MRCA (823), a buck born in the early 2000s, that may be considered an important source for the dissemination of the causal mutation associated with Mucopolysaccharidosis IIID in the Brazilian Anglo‐Nubian population.

Additionally, the genotype probabilities estimation resulted in the identification of 152 putative wild homozygous and eleven putative heterozygous for the causal mutation associated with Mucopolysaccharidosis IIID (GPI > 70%) and interestingly, it was possible to estimate with high probability (GPI = 100%) that the individual 823 is a putative heterozygous for the studied mutation, highlighting its role as an important source for the dissemination of the deleterious mutation into the Brazilian Anglo‐Nubian population (Table S5 and Figure S7).

In this study, the combination of genotyping and pedigree analysis enabled the estimation of high confidence putative genotypes representing an increase of ~55% of genotype information. Considering that goat can be considered a low‐value livestock species, implementation of any genotyping procedure must be considered in conjunction with additional strategies to reduce costs and then, the combination of genotyping with pedigree analysis can be considered an interesting alternative for the effective implementation of a program to control/eradicate mutations associated with genetic diseases (Bourneuf et al. 2017; Upperman et al. 2019; Mandal et al. 2021; Ablondi et al. 2022).

To deepen the knowledge into the genealogy of the causal mutation associated with Mucopolysaccharidosis IIID, the pedigrees of the identified MRCAs were extended including all available information, resulting in the addition of 775 new individuals and allowing the grouping of twelve out of sixteen heterozygous into one cluster (Figure S11) tracing back to a common ancestor (Table S6), a doe (1046) born in the 90s from a U.S. lineage. Even though this individual could not have the genotype predicted with high confidence, it can be suggested that it may have a central role in the process of introduction and dissemination of the causal mutation associated with Mucopolysaccharidosis IIID in the Brazilian Anglo‐Nubian population.

Finally, considering that the present study genotyped a limited number of individuals, it is not possible to determine that this pathway is the sole route for the introduction and dissemination of this allele in our country. Nevertheless, it can be considered an initial effort to pave the way to the implementation of a genetic disease eradication program in Brazil, enabling the proposition of initial strategies focused on testing Anglo‐Nubian individuals derived from this lineage to avoid the emergence of affected animals in Brazil (Marzanova et al. 2023).

## Author Contributions

**Flávia Caroline Moreira Bezerra:** formal analysis, investigation, writing – review and editing. **Lívia Samaniely Batista Pereira Belli:** investigation, writing – review and editing. **Joane Maíra Cavalcante Braga Novais:** investigation, writing – review and editing. **Adriana Mércia Guaratini Ibelli:** methodology, resources, writing – review and editing. **Cássia Regina Oliveira Santos:** investigation, writing – original draft, writing – review and editing. **Gisele Veneroni Gouveia:** conceptualization, methodology, resources, writing – review and editing. **Alexandre Tadeu Mota Macedo:** conceptualization, resources, writing – review and editing. **João José de Simoni Gouveia:** conceptualization, methodology, formal analysis, supervision, project administration, funding acquisition, writing – original draft, writing – review and editing.

## Funding

This study was supported by Fundação de Amparo à Ciência e Tecnologia do Estado de Pernambuco—FACEPE (grant numbers APQ‐1254‐5.05/22, APQ‐0108‐5.07/23) and Coordenação de Aperfeiçoamento de Pessoal de Nível Superior—Brasil (CAPES)—Finance Code 001.

## Conflicts of Interest

The authors declare no conflicts of interest.

## Supporting information


**Figure S1:** Validation, using synthetic DNA fragments (gBlocks), of the real time PCR test based on hydrolysis probes (Taqman) developed in this study to identify the causal mutation associated with Mucopolysaccharidosis IIID in goats. (A) Allelic discrimination plot. Light blue dot indicates the nontemplate control, red dots indicate homozygous control for the wild allele (CC), green dots indicate heterozygous control (CT), and yellow dots represent homozygous control for the mutation (TT); (B) Amplification curves for the wild allele homozygous control; (C) Amplification curves for the heterozygous control; (D) Amplification curves for the mutation homozygous control.
**Figure S2:** Validation, using synthetic DNA fragments (gBlocks), of the real time PCR test based on hydrolysis probes (Taqman) developed in this study to identify the causal mutation associated with Beta‐Mannosidosis in goats. (A) Allelic discrimination plot. Light blue dot indicates the nontemplate control, red dots indicate homozygous control for the wild allele (GG), green dots indicate heterozygous control (G/delG), and yellow dots represent homozygous control for the mutation (delG/delG); (B) Amplification curves for the wild allele homozygous control; (C) Amplification curves for the heterozygous control; (D) Amplification curves for the mutation homozygous control.
**Figure S3:** Electropherogram of the partial sequence of GNS gene to validate the genotypes identified by the real time PCR. Samples 34, 89, 12, 15, 61 and 16 were confirmed as heterozygous for the mutation associated with Mucopolysaccharidosis IIID (CT) and sample 05 was confirmed as homozygous (CC) for the wild allele.
**Figure S4:** Electropherogram of the partial sequence of GNS gene to validate the genotypes identified by the real time PCR. Samples 171, 179, 172, and 149 were confirmed as heterozygous for the mutation associated with Mucopolysaccharidosis IIID (CT) and samples 90, 101 and 112 were confirmed as homozygous (CC) for the wild allele.
**Figure S5:** Electropherogram of the partial sequence of GNS gene to validate the genotypes identified by the real time PCR. Samples 217, 284, 202, 215, 201 and 214 were confirmed as heterozygous for the mutation associated with Mucopolysaccharidosis IIID (CT) and sample 216 was confirmed as homozygous (CC) for the wild allele.
**Figure S6:** Electropherogram of the partial sequence of MANBA gene to validate the genotypes identified by the real time PCR. Samples 105, 06 and 15 were confirmed as homozygous (GG) for the wild allele.
**Figure S7:** Pedigree of six heterozygous individuals for the causal mutation associated with Mucopolysaccharidosis IIID tracing back to a common ancestor (823). Blue symbols represent genotyped and red symbols represent ungenotyped animals. Filled symbols (left) indicate heterozygous genotypes identified by real time PCR and filled symbols with diagonal bars (right) indicate heterozygous genotypes predicted with the approach described in Kerr and Kinghorn (1996) with GPI > 70%. Symbols not filled represent wild homozygous individuals and “?” represent nonidentified genotype for the studied mutation.
**Figure S8:** Pedigree of two heterozygous individuals for the causal mutation associated with Mucopolysaccharidosis IIID tracing back to common ancestors (729 and 836). Blue symbols represent genotyped and red symbols represent ungenotyped animals. Filled symbols (left) indicate heterozygous genotypes identified by real time PCR and filled symbols with diagonal bars (right) indicate heterozygous genotypes predicted with the approach described in Kerr and Kinghorn (1996) with GPI > 70%. Symbols not filled represent wild homozygous individuals and “?” represent nonidentified genotype for the studied mutation.
**Figure S9:** Pedigree of two heterozygous individuals for the causal mutation associated with Mucopolysaccharidosis IIID tracing back to a common ancestor (1052). Blue symbols represent genotyped and red symbols represent ungenotyped animals. Filled symbols (left) indicate heterozygous genotypes identified by real time PCR and filled symbols with diagonal bars (right) indicate heterozygous genotypes predicted with the approach described in Kerr and Kinghorn (1996) with GPI > 70%. Symbols not filled represent wild homozygous individuals and “?” represent nonidentified genotype for the studied mutation.
**Figure S10:** Pedigree of two heterozygous individuals for the causal mutation associated with Mucopolysaccharidosis IIID tracing back to a common ancestor (805). Blue symbols represent genotyped and red symbols represent ungenotyped animals. Filled symbols (left) indicate heterozygous genotypes identified by real time PCR and filled symbols with diagonal bars (right) indicate heterozygous genotypes predicted with the approach described in Kerr and Kinghorn (1996) with GPI > 70%. Symbols not filled represent wild homozygous individuals and “?” represent nonidentified genotype for the studied mutation.
**Figure S11:** Heatmap of the kinship coefficients from heterozygous animals for the mutation associated with Mucopolysaccharidosis IIID identified in this study after extension of the pedigrees of the MRCAs identified.
**Table S1:** Distribution of animals used in this study.
**Table S2:** Primers and probes designed to genotype causal mutations associated with Mucopolysaccharidosis IIID and Beta‐Mannosidosis.
**Table S3:** Sequencing primers designed to genotype causal mutations associated with Mucopolysaccharidosis IIID and Beta‐Mannosidosis.
**Table S4:** Description of animals confirmed heterozygous for the causal mutation associated with Mucopolysaccharidosis IIID identified in this study.
**Table S5:** Results from MRCA analysis.
**Table S6:** Results from MRCA analysis after extension of the pedigrees of the MRCAs identified.

## Data Availability

The data that support the findings of this study are available on request from the corresponding author. The data are not publicly available due to privacy or ethical restrictions.
