# Supplementary material for: Mucopolysaccharidosis IIID and Beta‐Mannosidosis in Brazilian Anglo‐Nubian Goats: Molecular and Genealogical Insights for the Development and Implementation of a Genetic Disease Eradication Program
Source: Anim Genet. 2026 Apr 22;57:e70107. doi: 10.1002/age.70107 (PMC13101034; doi:10.1002/age.70107)
Supplement: Supplementary file 1 — Figure S1: Validation, using synthetic DNA fragments (gBlocks), of the real time PCR test based on hydrolysis probes (Taqman) developed in this study to identify the causal mutation associated with Mucopolysaccharidosis IIID in goats. (A) Allelic discrimination plot. Light blue dot indicates the nontemplate control, red dots indicate homozygous control for the wild allele (CC), green dots indicate heterozygous control (CT), and yellow dots represent homozygous control for the mutation (TT); (B) Amplification curves for the wild allele homozygous control; (C) Amplification curves for the heterozygous control; (D) Amplification curves for the mutation homozygous control. Figure S2: Validation, using synthetic DNA fragments (gBlocks), of the real time PCR test based on hydrolysis probes (Taqman) developed in this study to identify the causal mutation associated with Beta‐Mannosidosis in goats. (A) Allelic discrimination plot. Light blue dot indicates the nontemplate control, red dots indicate homozygous control for the wild allele (GG), green dots indicate heterozygous control (G/delG), and yellow dots represent homozygous control for the mutation (delG/delG); (B) Amplification curves for the wild allele homozygous control; (C) Amplification curves for the heterozygous control; (D) Amplification curves for the mutation homozygous control. Figure S3: Electropherogram of the partial sequence of GNS gene to validate the genotypes identified by the real time PCR. Samples 34, 89, 12, 15, 61 and 16 were confirmed as heterozygous for the mutation associated with Mucopolysaccharidosis IIID (CT) and sample 05 was confirmed as homozygous (CC) for the wild allele. Figure S4: Electropherogram of the partial sequence of GNS gene to validate the genotypes identified by the real time PCR. Samples 171, 179, 172, and 149 were confirmed as heterozygous for the mutation associated with Mucopolysaccharidosis IIID (CT) and samples 90, 101 and 112 were confirmed as homozygous (CC) for the w [file AGE-57-0-s001.docx]

**SUPPLEMENTARY MATERIAL**

**Summary**

[**1.** **Detailed materials and methods** 2](#_Toc213898905)

[**1.1.** **Ethical aspects** 2](#_Toc213898906)

[**1.2.** **Sample collection and DNA extraction** 2](#_Toc213898907)

[**1.3.** **gBlocks™ Gene Fragments design and synthesis** 2](#_Toc213898908)

[**1.4.** **Primers and probes design** 3](#_Toc213898909)

[**1.5.** **Real time PCR tests based on hydrolysis probes (Taqman®)** 3](#_Toc213898910)

[**1.6.** **Validation of the real time PCR genotyping by Sanger sequencing** 4](#_Toc213898911)

[**1.7.** **Pedigree analysis** 4](#_Toc213898912)

[**2.** **Supplementary Figures** 5](#_Toc213898913)

[**3.** **Supplementary Tables** 16](#_Toc213898914)

[**4.** **Supplementary References** 22](#_Toc213898915)

1. **Detailed materials and methods**

- 1. **Ethical aspects**

This study was authorized by the Ethics Committee for the Use of Animals from the Federal University of Vale do São Francisco (CEUA/UNIVASF) under authorization number 0002/290922. All samples were collected and analyses performed after obtaining written informed consent from owners.

- 1. **Sample collection and DNA extraction**

A total of two hundred ninety-five (n=295) individuals from Anglo-Nubian goat breed (registered in the herdbook of the Brazilian Goat Breeders Association) belonging to 13 herds from Pernambuco, Bahia, Ceará and Piauí states (Brazil) were used in this study (Supplementary Table *1*).

Blood samples were collected through venipuncture using vacuum collection tubes with K_2_ EDTA and DNA extraction was performed through a salting out procedure proposed by Regitano et al. (2007). Quality and quantity verification of DNA was assessed using 2% agarose gel electrophoresis (80 V, 120 mA, 2 h) stained with ethidium bromide and by spectrophotometry using NanoDrop. DNA was diluted to a final concentration of 40 ng/μL and stored at -20ºC until utilization.

- 1. **gBlocks™ Gene Fragments design and synthesis**

Two synthetic DNA fragments (gBlocks) were synthesized by IDT Integrated DNA Technologies® to be used as reaction controls for the real time PCR genotyping procedures. One fragment consisted of the partial sequence of the goat *GNS* and *MANBA* genes harboring the causative mutations studied and its flanking sequences (~75bp upstream and downstream). The other fragment consisted of the same sequences described above harboring the wild alleles. Synthetic DNA fragments were resuspended following manufacturer’s protocol that consisted in the addition of 1 X TE (10 mM, pH 8.0 Tris HCl; 1 mM, pH 8.0 EDTA) followed by an incubation at 50 ºC for 20 min. After this step, control solutions were diluted to a final concentration of 0,4 pg/µL. Heterozygote controls were obtained pooling the two gBlocks synthesized in a 1:1 proportion.

- 1. **Primers and probes design**

To develop molecular tests based on hydrolysis probes (Taqman-MGB) for the causal mutations associated with Mucopolysaccharidosis IIID and Beta-Mannosidosis, two sets of primers and probes were designed using public reference sequences for caprine *GNS* (NC_030812.1) and *MANBA* (NC_030813.1) genes deposited in NCBI. The design and quality analysis of the primers and probes was performed with Primer3plus (Untergasser et al., 2007), Edesign (Kimura et al., 2016), OligoAnalyser (Owczarzy et al. 2008) and PrimerExpress (Life Technologies Corporation, 2011) and the selected sets were synthetized by ThermoFisherScientific. Sequencing primers to validate the real time genotyping procedure were designed using the same reference sequences described above with the use of Primer3plus (Untergasser et al., 2007) and OligoAnalyzer (Owczarzy et al. 2008) tools and synthetized by Exxtend company in Brazil.

- 1. **Real time PCR tests based on hydrolysis probes (Taqman®)**

Primers and probes designed in this study (*Supplementary Table 2*) were used to genotype individuals for causal mutations associated with Mucopolysaccharidosis IIID and Beta-Mannosidosis. Real time PCR reactions were performed using GoTaq Probe qPCR e RT-qPCR Systems (Promega®) or TaqPath ProAmp Master Mix (ThermoFisherScientific) kits. Reactions using GoTaq Probe qPCR e RT-qPCR Systems (Promega) were done in a final volume of 10 µL using 5 µL of GoTaq Probe qPCR High 2X, 0.5 µL of 20 x Assay Working Stock, 1 µL of DNA (40 ng/µL) and 3.5 µL of nuclease free water. The amplification steps were: (1) 95 °C, 2 min; (2) 95 °C, 1 s; (3) 60.0 °C, 30 s; (4) Repeat steps 2–3 40X. Reactions using TaqPath ProAmp Master Mix da ThermoFisherScientific® were done in a final volume of 10 µL using 5 µL of TaqPath ProAmp Master Mix, 0.5 µL of 20 x Assay Working Stock, 1 µL of DNA (40 ng/µL) and 3.5 µL of nuclease free water. The amplification steps were: (1) 95 °C, 2 min; (2) 95 °C, 3 s; (3) 60.0 °C, 30 s; (4) Repeat steps 2–3 40X.

- 1. **Validation of the real time PCR genotyping by Sanger sequencing**

Partial amplification of the *GNS* and *MANBA* genes using sequencing primers developed in this study (*Supplementary Table 3*) was performed using TopTaq DNA Polymerase (QIAGEN) enzyme.

A total of 21 samples (16 genotyped by real time PCR as heterozygotes and 5 as homozygous for the wild allele) were used for partial amplification of *GNS* gene and three samples (genotyped by real time PCR as homozygous for the wild allele) were used for partial amplification of *MANBA* gene. Both genes were partially amplified with a reaction consisting of 5 X Toptaq PCR Master Mix, 2 µM each primer and 80 ng DNA in a final volume of 50 μL. The amplification steps were: (1) 95 °C, 05 min; (2) 95 °C, 45 s; (3) 57.8 °C, 45 s; (4) 72 °C, 45 s; (5) Repeat steps 2–4 30X; (6) 72 °C, 10 min. All samples were sent to a private company for bidirectional Sanger sequencing and the raw electropherograms were processed with Phred/Phrap/Consed suite (Ewing and Green, 1998; Gordon et al., 1998) and then, visual inspected to confirm the genotypes for the causal mutations studied.

- 1. **Pedigree analysis**

Genealogical information was retrieved from the Brazilian Goat Breeders Association database (Associação Brasileira de Criadores de Caprinos, 2025) using the unique registry identification of each animal. Pedigree visualization and analysis were performed using the R packages kinship2 (Sinnwell et al., 2014) and Pedigree (Coster, 2013) and Endog v4.8 software (Gutiérrez and Goyache, 2005). Additionally, the approach described in Kerr and Kinghorn (1996) was used to estimate the genotype probabilities from ungenotyped individuals using Geneprob software (http://www-personal.une.edu.au/∼bkinghor/geneprob.htm), and the R package Genlib (Gauvin et al., 2015) was used to identify the most recent common ancestors (MRCA) of heterozygous individuals for the mutations studied.

1. **Supplementary Figures**


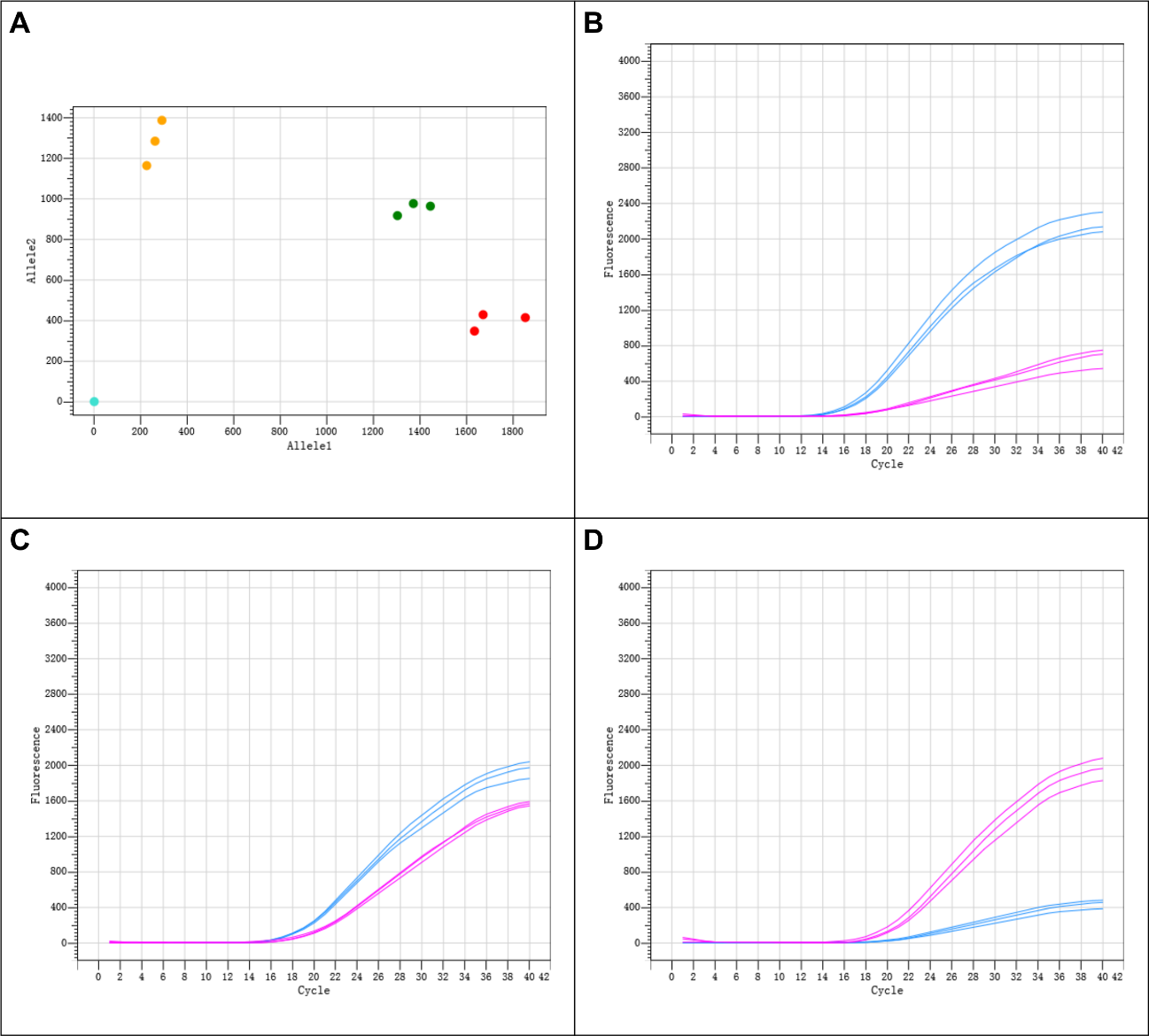


Supplementary Figure 1. Validation, using synthetic DNA fragments (gBlocks™), of the real time PCR test based on hydrolysis probes (Taqman®) developed in this study to identify the causal mutation associated with Mucopolysaccharidosis IIID in goats. A) Allelic discrimination plot. Light blue dot indicates the nontemplate control, red dots indicate homozygous control for the wild allele (CC), green dots indicate heterozygous control (CT), and yellow dots represent homozygous control for the mutation (TT); B) Amplification curves for the wild allele homozygous control; C) Amplification curves for the heterozygous control; D) Amplification curves for the mutation homozygous control.


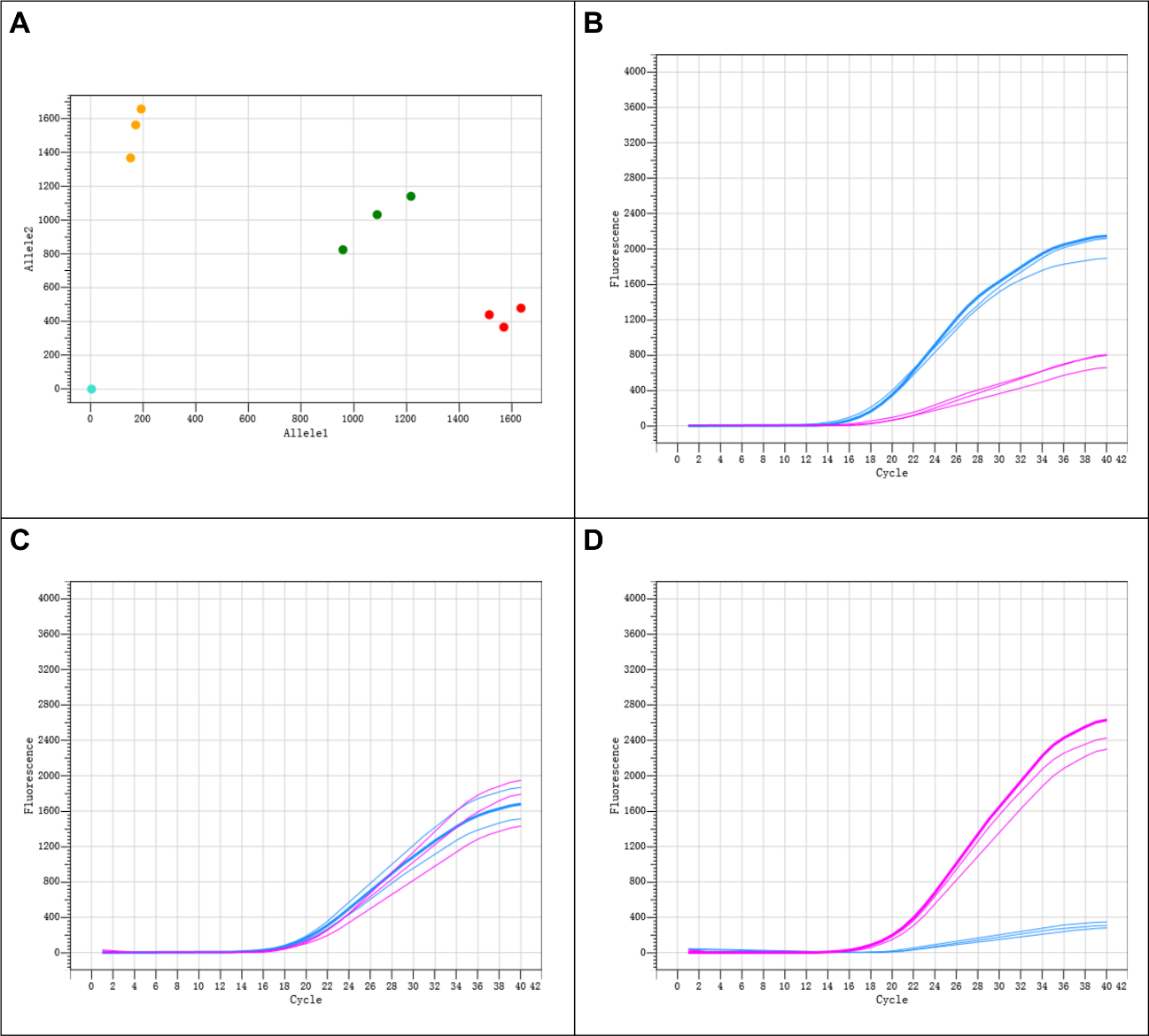


Supplementary Figure 2. Validation, using synthetic DNA fragments (gBlocks™), of the real time PCR test based on hydrolysis probes (Taqman®) developed in this study to identify the causal mutation associated with Beta-Mannosidosis in goats. A) Allelic discrimination plot. Light blue dot indicates the nontemplate control, red dots indicate homozygous control for the wild allele (GG), green dots indicate heterozygous control (G/delG), and yellow dots represent homozygous control for the mutation (delG/delG); B) Amplification curves for the wild allele homozygous control; C) Amplification curves for the heterozygous control; D) Amplification curves for the mutation homozygous control.


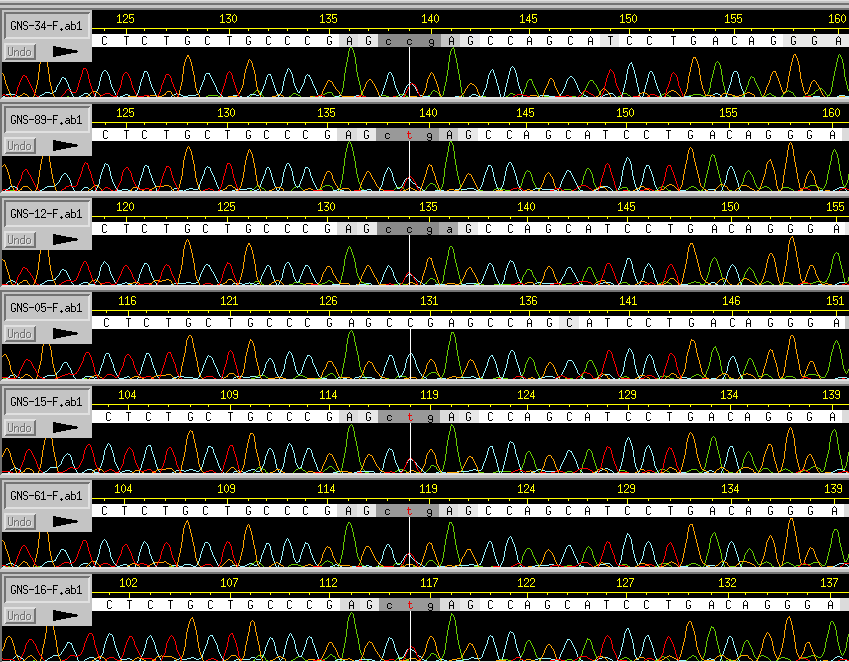


Supplementary Figure 3. Electropherogram of the partial sequence of GNS gene to validate the genotypes identified by the real time PCR. Samples 34, 89, 12, 15, 61 and 16 were confirmed as heterozygous for the mutation associated with Mucopolysaccharidosis IIID (CT) and sample 05 was confirmed as homozygous (CC) for the wild allele.


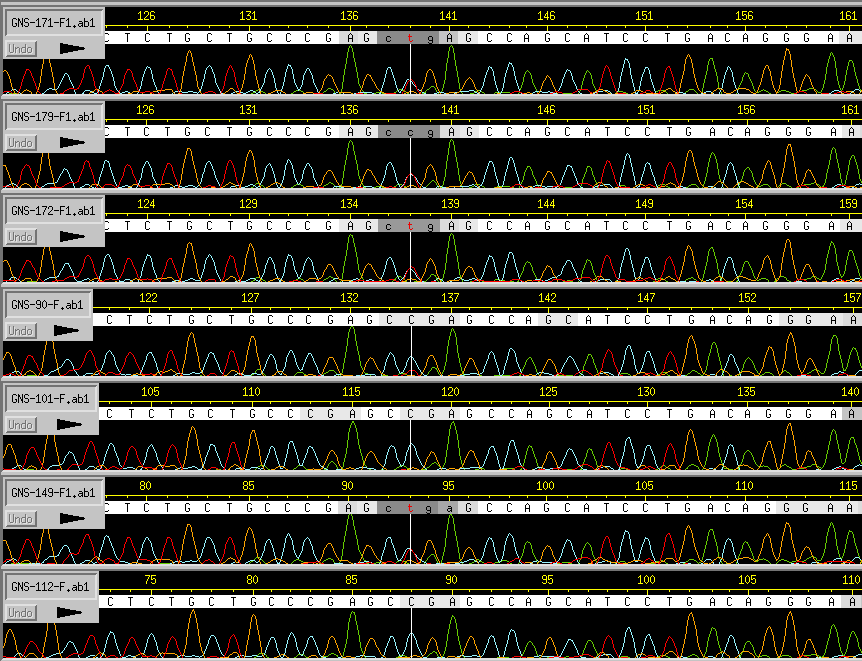


Supplementary Figure 4. Electropherogram of the partial sequence of GNS gene to validate the genotypes identified by the real time PCR. Samples 171, 179, 172, and 149 were confirmed as heterozygous for the mutation associated with Mucopolysaccharidosis IIID (CT) and samples 90, 101 and 112 were confirmed as homozygous (CC) for the wild allele.


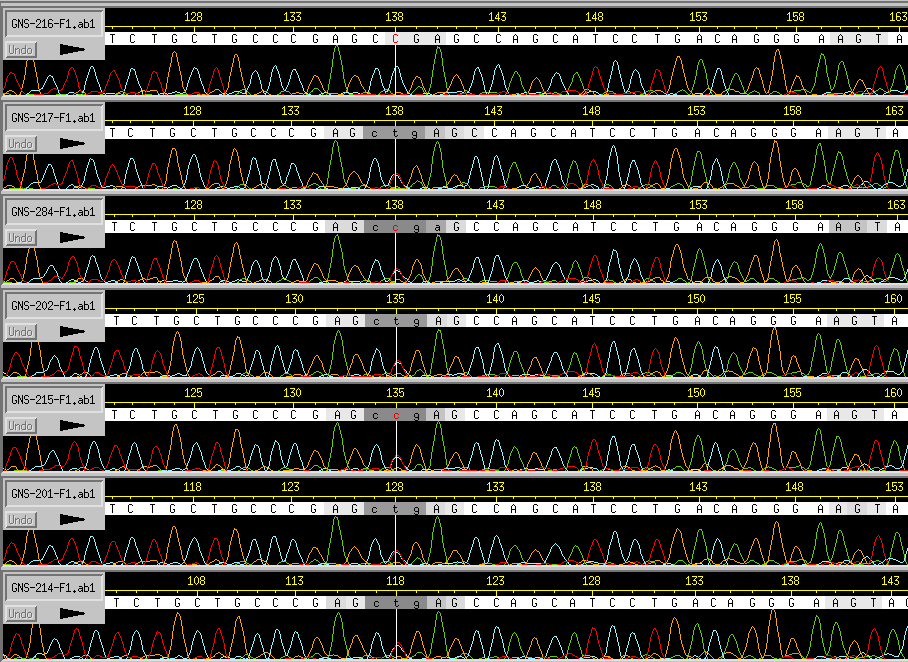


Supplementary Figure 5. Electropherogram of the partial sequence of GNS gene to validate the genotypes identified by the real time PCR. Samples 217, 284, 202, 215, 201 and 214 were confirmed as heterozygous for the mutation associated with Mucopolysaccharidosis IIID (CT) and sample 216 was confirmed as homozygous (CC) for the wild allele.


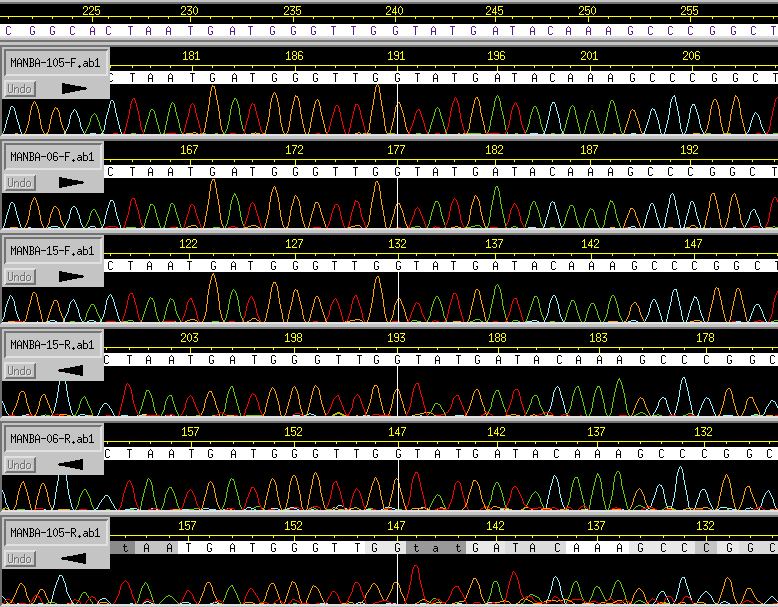


Supplementary Figure 6. Electropherogram of the partial sequence of MANBA gene to validate the genotypes identified by the real time PCR. Samples 105, 06 and 15 were confirmed as homozygous (GG) for the wild allele.


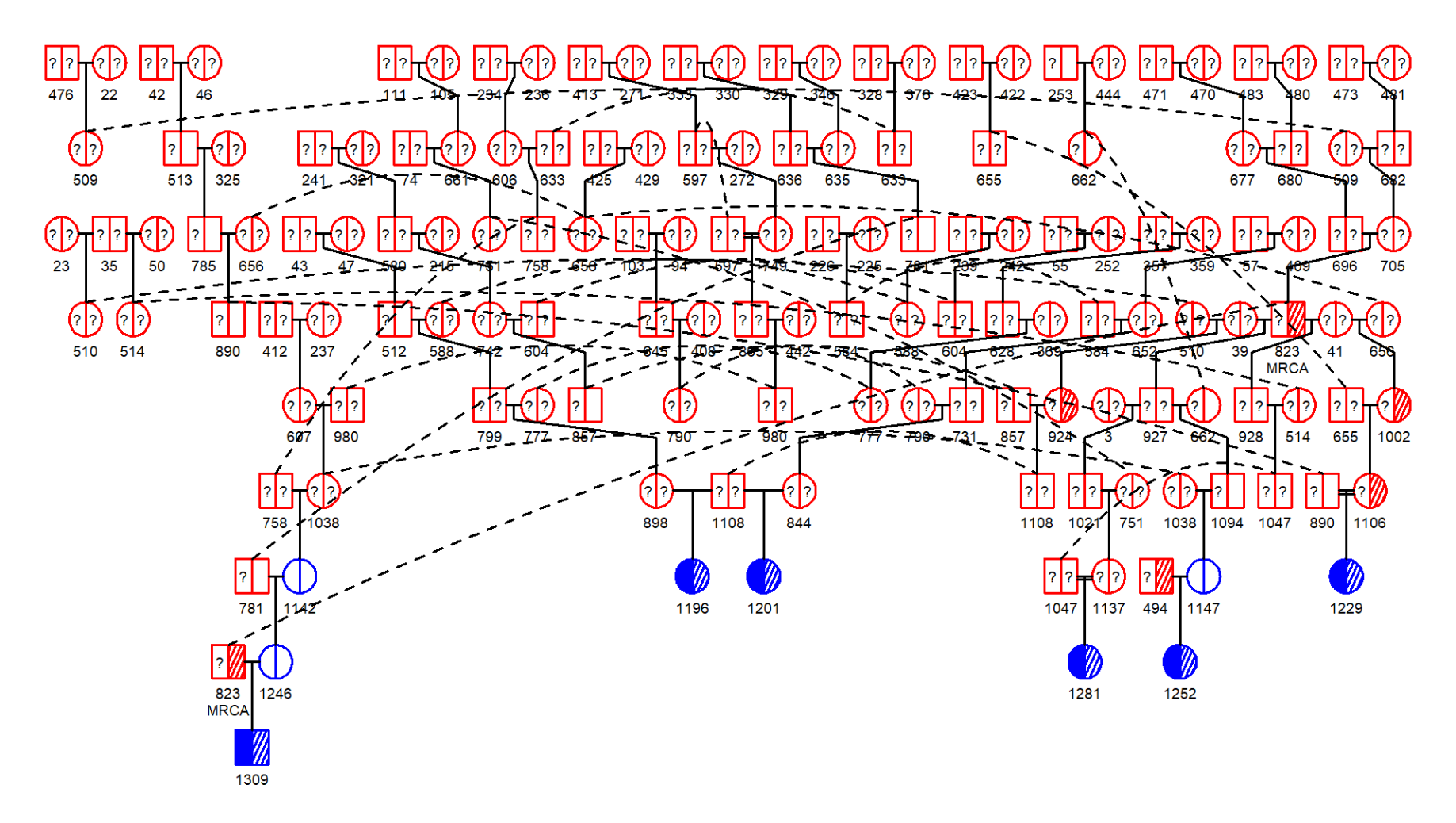


Supplementary Figure 7. Pedigree of six heterozygous individuals for the causal mutation associated with Mucopolysaccharidosis IIID tracing back to a common ancestor (823). Blue symbols represent genotyped and red symbols represent ungenotyped animals. Filled symbols (left) indicate heterozygous genotypes identified by real time PCR and filled symbols with diagonal bars (right) indicate heterozygous genotypes predicted with the approach described in Kerr and Kinghorn (1996) with GPI > 70%. Symbols not filled represent wild homozygous individuals and “?” represent nonidentified genotype for the studied mutation.


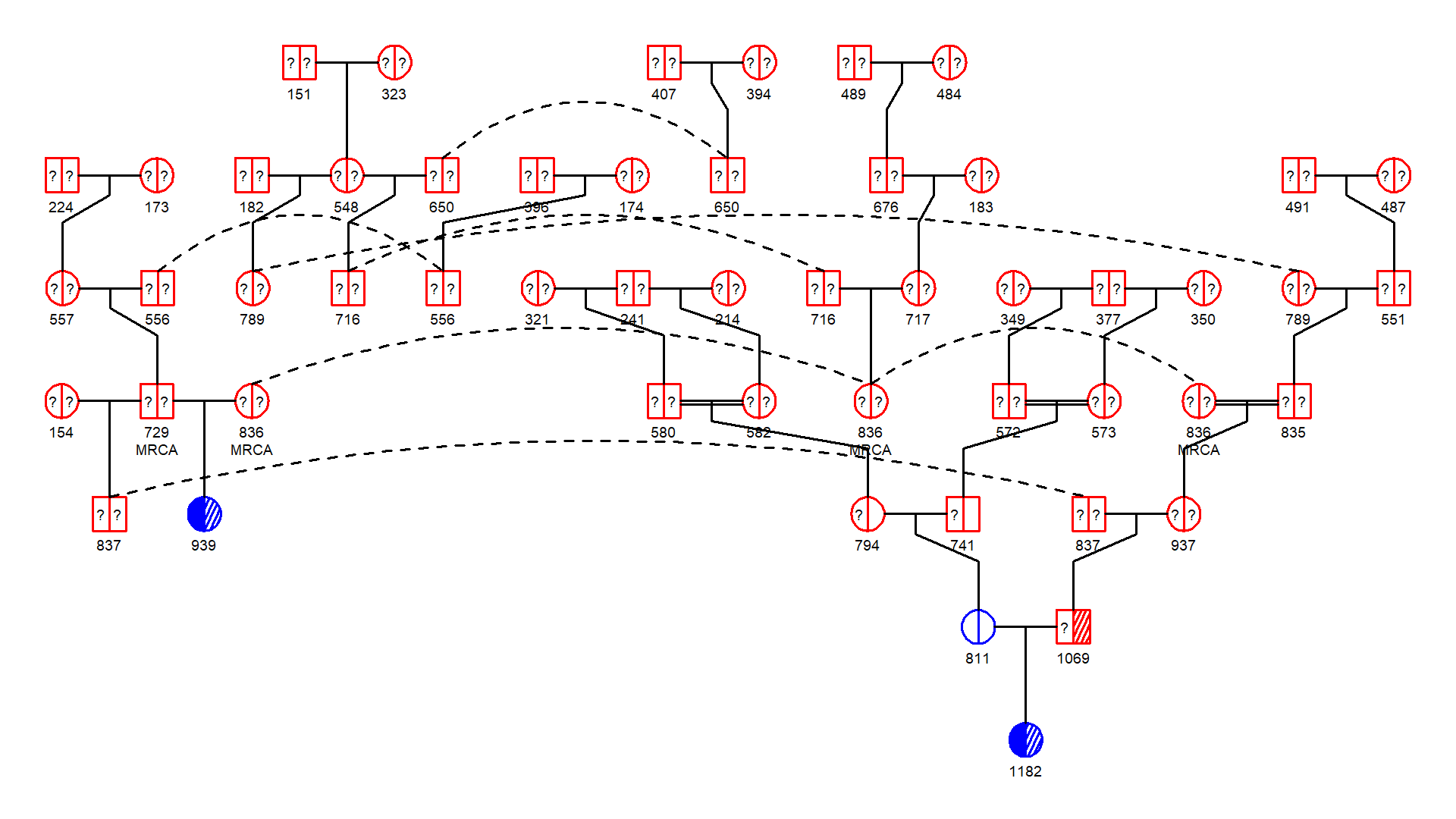


Supplementary Figure 8. Pedigree of two heterozygous individuals for the causal mutation associated with Mucopolysaccharidosis IIID tracing back to common ancestors (729 and 836). Blue symbols represent genotyped and red symbols represent ungenotyped animals. Filled symbols (left) indicate heterozygous genotypes identified by real time PCR and filled symbols with diagonal bars (right) indicate heterozygous genotypes predicted with the approach described in Kerr and Kinghorn (1996) with GPI > 70%. Symbols not filled represent wild homozygous individuals and “?” represent nonidentified genotype for the studied mutation.


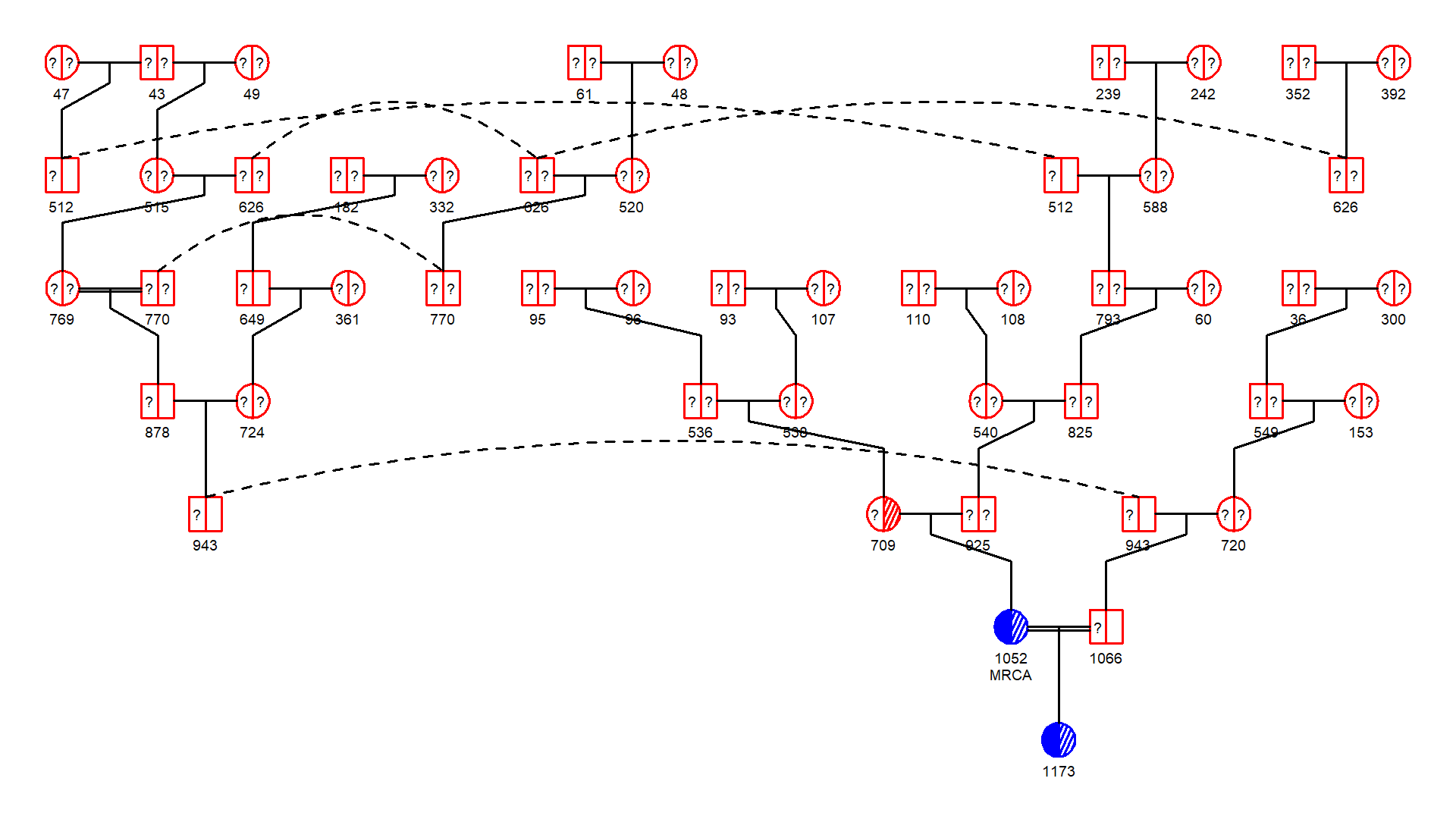


Supplementary Figure 9. Pedigree of two heterozygous individuals for the causal mutation associated with Mucopolysaccharidosis IIID tracing back to a common ancestor (1052). Blue symbols represent genotyped and red symbols represent ungenotyped animals. Filled symbols (left) indicate heterozygous genotypes identified by real time PCR and filled symbols with diagonal bars (right) indicate heterozygous genotypes predicted with the approach described in Kerr and Kinghorn (1996) with GPI > 70%. Symbols not filled represent wild homozygous individuals and “?” represent nonidentified genotype for the studied mutation.


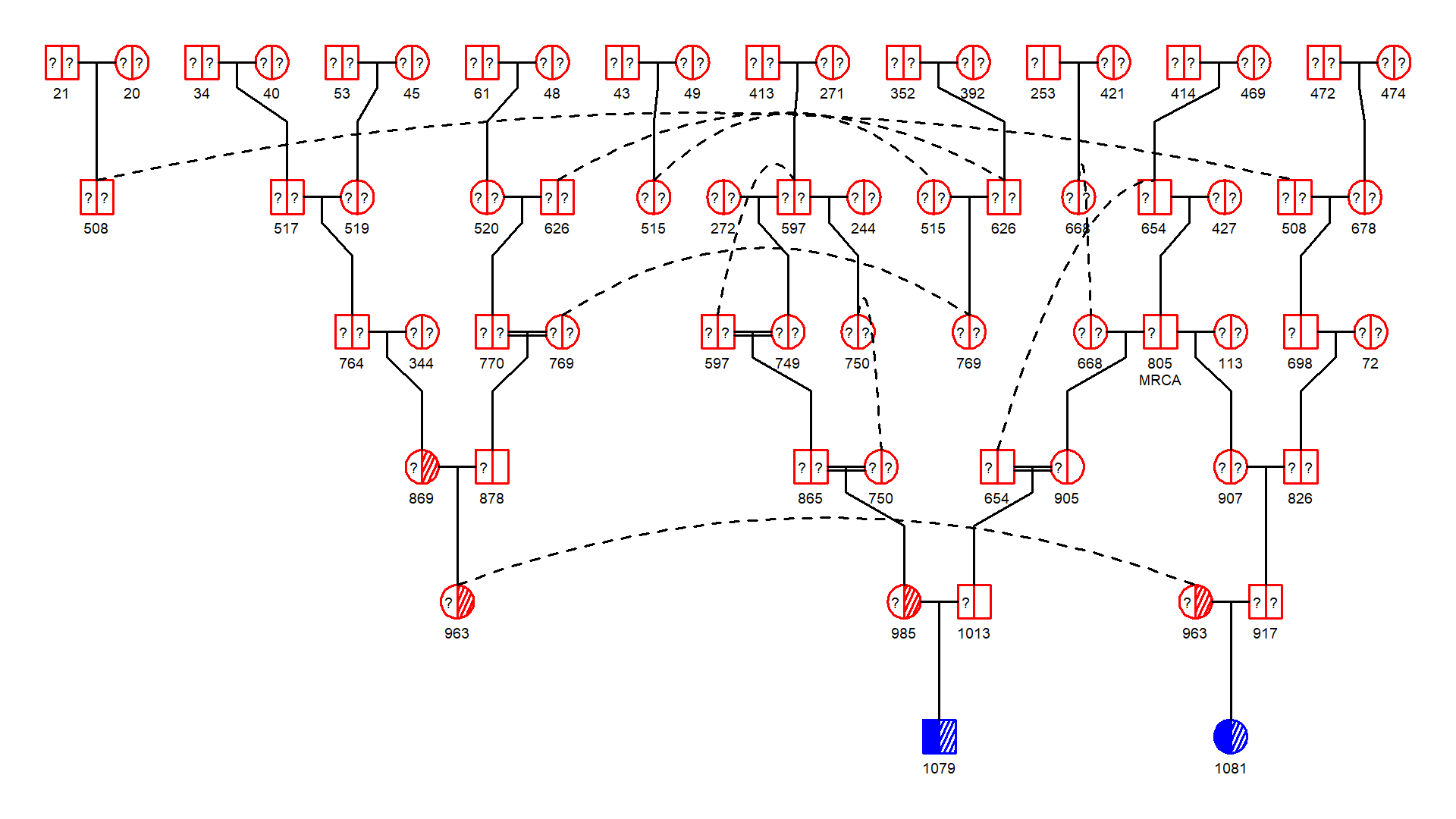


Supplementary Figure 10. Pedigree of two heterozygous individuals for the causal mutation associated with Mucopolysaccharidosis IIID tracing back to a common ancestor (805). Blue symbols represent genotyped and red symbols represent ungenotyped animals. Filled symbols (left) indicate heterozygous genotypes identified by real time PCR and filled symbols with diagonal bars (right) indicate heterozygous genotypes predicted with the approach described in Kerr and Kinghorn (1996) with GPI > 70%. Symbols not filled represent wild homozygous individuals and “?” represent nonidentified genotype for the studied mutation.


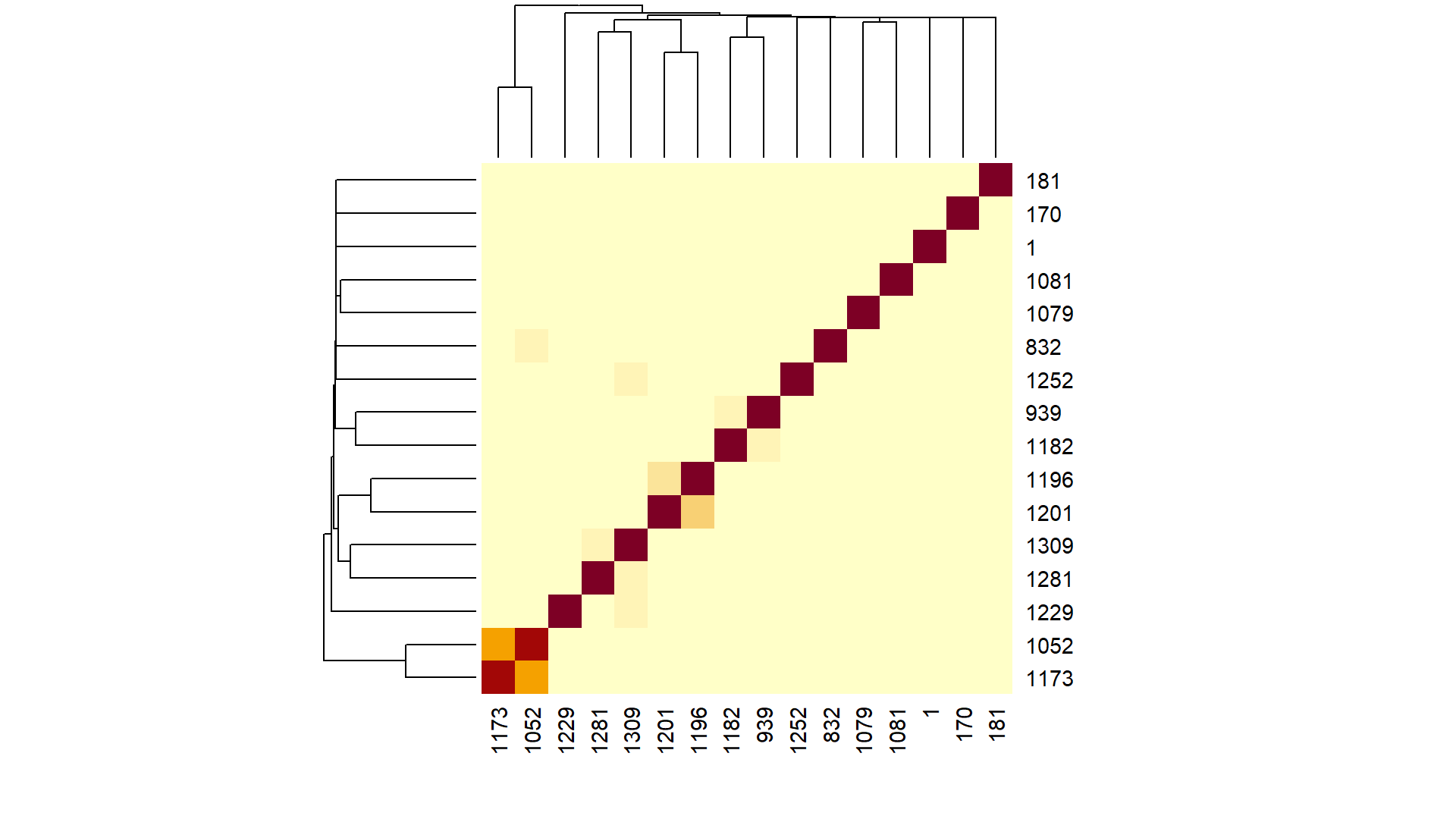


Supplementary Figure 11. Heatmap of the kinship coefficients from heterozygous animals for the mutation associated with Mucopolysaccharidosis IIID identified in this study after extension of the pedigrees of the MRCAs identified.

1. **Supplementary Tables**

Supplementary Table 1. Distribution of animals used in this study.

| Herd | Location (state) | Sampled individuals | Number of males (M) and females (F) |
| --- | --- | --- | --- |
| 1 | Bahia | 33 | 07 M and 26 F |
| 2 | Bahia | 12 | 02 M and 10 F |
| 3 | Bahia | 26 | 03 M and 23 F |
| 4 | Bahia | 42 | 09 M and 33 F |
| 5 | Bahia | 36 | 06 M and 30 F |
| 6 | Pernambuco | 42 | 07 M and 35 F |
| 7 | Bahia | 06 | 02 M and 04 F |
| 8 | Ceará | 22 | 05 M and 17 F |
| 9 | Piauí | 03 | 0 M and 03 F |
| 10 | Pernambuco | 15 | 5 M and 10 F |
| 11 | Pernambuco | 05 | 0 M and 05 F |
| 12 | Pernambuco | 24 | 02 M and 22 F |
| 13 | Ceará | 29 | 02 M and 27 F |

Supplementary Table 2. Primers and probes designed to genotype causal mutations associated with Mucopolysaccharidosis IIID and Beta-Mannosidosis

| Gene | Identification | Fluorophore | Sequence (5’-3’) | Allele |
| --- | --- | --- | --- | --- |
| *GNS* | Forward Primer | - | TGCCAAGTGCTCTCTGCTG | - |
|  | Reverse Primer | - | GTTCCTGGATCTTCTGCCAAG | - |
|  | Wild Allele Probe | FAM | AGCCGAGCCAGCAT | C |
|  | Mutant Allele Probe | VIC | CCCGAGCTGAGCCA | T |
| *MANBA* | Forward Primer | - | ATCCCTCCATCATCACATGG | - |
|  | Reverse Primer | - | CGATCGTTCGGATGTTTTTC | - |
|  | Wild Allele Probe | FAM | ATGGGTTGGTATGATACA | G |
|  | Mutant Allele Probe | VIC | ATGGGTTGTATGATACAA | delG |

Supplementary Table 3. Sequencing primers designed to genotype causal mutations associated with Mucopolysaccharidosis IIID and Beta-Mannosidosis.

| Gene | Identification | Sequence (5’-3’) |
| --- | --- | --- |
| *GNS* | Forward Primer | AAGCAGCTCTGAAGTGTGTCA |
|  | Reverse Primer | GCCAGCTACCCGAAAACCCT |
| *MANBA* | Forward Primer | CTTCCCACATGTAACATTACTGCTC |
|  | Reverse Primer | GAGGCACACTGAGTTTGGGTA |

Supplementary Table 4. Description of animals confirmed heterozygous for the causal mutation associated with Mucopolysaccharidosis IIID identified in this study

| Animal | Lab ID | Father | Mother | Sex | Herd | Location (state) | F |
| --- | --- | --- | --- | --- | --- | --- | --- |
| 832 | CHAN0015 | 708 | 707 | Female | 1 | Bahia | 0,00% |
| 1052 | CHAN0016 | 925 | 709 | Female | 1 | Bahia | 0,00% |
| 1173 | CHAN0012 | 1066 | 1052 | Female | 1 | Bahia | 0,05% |
| 1182 | CHAN0034 | 1069 | 811 | Female | 2 | Bahia | 0,00% |
| 1196 | CHAN0061 | 1108 | 898 | Female | 3 | Bahia | 0,00% |
| 1229 | CHAN0089 | 890 | 1106 | Female | 4 | Bahia | 6,25% |
| 939 | CHAN0149 | 729 | 836 | Female | 5 | Bahia | 0,00% |
| 1 | CHAN0172 | ? | ? | Female | 6 | Pernambuco | 0,00% |
| 1252 | CHAN0179 | 494 | 1147 | Female | 6 | Pernambuco | 0,00% |
| 1309 | CHAN0171 | 823 | 1246 | Male | 6 | Pernambuco | 0,00% |
| 181 | CHAN0214 | ? | ? | Female | 8 | Ceará | 0,00% |
| 1079 | CHAN0217 | 1013 | 985 | Male | 8 | Ceará | 0,00% |
| 1081 | CHAN0215 | 917 | 963 | Female | 8 | Ceará | 0,00% |
| 1201 | CHAN0202 | 1108 | 844 | Female | 8 | Ceará | 0,00% |
| 1281 | CHAN0201 | 1047 | 1137 | Female | 8 | Ceará | 1,56% |
| 170 | CHAN0284 | ? | ? | Female | 13 | Ceará | 0,00% |

Supplementary Table 5. Results from MRCA analysis.

| MRCA | Heterozygous descendants | Genotype |
| --- | --- | --- |
| 823 | 1196, 1201, 1229, 1252, 1281 and 1309 | Heterozygous (CT) * |
| 729 and 836 | 939 and 1182 | - |
| 805 | 1079 and 1081 | Wild homozygous (CC) * |
| 1052 | 1052 and 1173 | Heterozygous (CT) ** |

*Predicted genotype for the causal mutation associated to Mucopolysaccharidosis IIID based on the approach described in Kerr and Kinghorn (1996) with GPI > 70%. **Genotyped in this study.

Supplementary Table 6. Results from MRCA analysis after extension of the pedigrees of the MRCAs identified.

| MRCA | Heterozygous descendants | Genotype |
| --- | --- | --- |
| 1046 | 1309, 1196, 1201, 1281, 1252, 939, 1182, 1079, 1081, 1052, 1173 and 1229 | - |

1. **Supplementary References**

Ewing, B., Green, P., 1998. Base-Calling of Automated Sequencer Traces Using Phred. II. Error Probabilities. Genome Research. 8, 186-194. https://doi.org/10.1101/gr.8.3.186

Gordon, D., Abajian, C. & Green, P., 1998. Consed: A Graphical Tool for Sequence Finishing. Genome Research. 8, 195-202. https://doi.org/10.1101/gr.8.3.195

Kimura, Y., Soma, T., Kasahara, N., Delobel, D., Hanami, T., Tanaka, Y., Hoon, M.J.L. de., Hayashizaki, Y., Usui, K. & Harbers, M., 2016. Edesign: Primer and Enhanced Internal Probe Design Tool for Quantitative PCR Experiments and Genotyping Assays. Plos One. 11(3). https://doi.org/10.1371/journal.pone.0146950

Owczarzy, R., Tataurov, A.V., Wu, Y., Manthey, J.A., McQuisten, K.A., Almabrazi, H.G., Pedersen, K.F., Lin, Y., Garretson, J., McEntaggart, N.O., Sailor, C.A., Dawson, R.B. & Peek, A.S., 2008. IDT SciTools: a suite for analysis and design of nucleic acid oligomers. Nucleic Acids Research. 36, 163-169. https://doi.org/10.1093/nar/gkn198

Regitano, l.C.De A., Niciura, S.C.M., Ibelli, A.M.G., Gouveia, J.J. de S., 2007. Protocolos de Biologia Molecular Aplicada à Produção Animal. In: Gouveia, J.J.S., Regitano, L.C.A. Extração de DNA. Firts edition. São Carlos, São Paulo.

Untergasser, A., Cutcutache, I., Koressaar, T., Ye, J., Faircloth, B.C., Remm, M. & Rozen, S.G., 2012. Primer3—new capabilities and interfaces. Nucleic Acids Res. 40, 115-127. https://doi.org/10.1093/nar/gks596
